# Understanding what Australians find fearful and hopeful about climate change through qualitative approaches

**DOI:** 10.1371/journal.pone.0339306

**Published:** 2026-01-07

**Authors:** Matthew I. Mackay, Anna Klas, Emily J. Kothe, Kate Barford, Julian W. Fernando, Mathew Ling

**Affiliations:** 1 Deakin University, School of Psychology, Melbourne, Australia; 2 La Trobe University, Melbourne, Australia; 3 Neami National, Preston, Victoria; Queensland University of Technology, AUSTRALIA

## Abstract

Future-oriented emotional appeals, such as fear or hope, may be more effective in increasing climate action when they reflect the specific fears and hopes of the target population. However, qualitative evidence on what people find uniquely fearful and uniquely hopeful about climate change remains limited. To address this gap, an online qualitative survey asked 299 Australians (*M*_*age*_ = 33.09, *SD*_*age*_ = 12.14) to identify what they found fearful and hopeful about climate change. Through inductive thematic analysis, three themes reflected Australians’ fear: (1) ‘Change and Instability’, (2) ‘Inaction and Negligence by Government, Large Corporations, and Others’, and (3) ‘Intergenerational Impacts and Legacy’. Additionally, three themes reflected Australians’ hope: (1) ‘Changing Attitudes and Changing Pro-environmental Habits’, (2) ‘Progress, Technology, Sustainability, and Innovation’, and (3) ‘An Opportunity for Change’. While some elements of what Australians find fearful or hopeful may be unique (e.g., bushfires), others (e.g., intergenerational impacts) align with global concerns. These insights offer valuable guidance for designing interventions that aim to foster fear and hope to promote climate action.

## Introduction

Given the rapid environmental and societal transformations required to avoid the catastrophic consequences anticipated by climate change [1], uncertainty about future circumstances on Earth is increasingly pervasive [2,3]. The uncertainty people experience because of climate change is a psychological barrier to consistent pro-environmental behaviour [4]. Additionally, people may underestimate future environmental risks [5,6], such as the frequency and intensity of extreme weather events and their societal impacts (e.g., climate refugees). As individuals face these potential yet likely impacts, they experience a range of positive and negative emotions (e.g., fear, guilt, pride, hope) [7,8]. While general affect and specific emotions have motivational components and predict climate actions [7–10], future-oriented emotions may be particularly effective in promoting climate action due to their alignment with the temporal and uncertain nature of climate change [11].

Two future-oriented emotions commonly employed in behavioural interventions (e.g., persuasive messages) are fear and hope [9,12–14]. While fear is characterised by the belief that harmful and undesirable outcomes (e.g., an increase in extreme weather events) may occur in the future if no action to mitigate the threat (e.g., reduce carbon emissions) is taken [15], hope is characterised by the belief that positive and desirable outcomes (e.g., a clean and renewable energy future) may occur in the future if action to mitigate the threat (e.g., climate change) is taken [15]. Although commonly employed, current research, as well as its applications by climate communicators, climate activists and government agencies [16,17], is limited by the lack of qualitative evidence identifying what people find uniquely fearful and hopeful about climate change. This limitation is significant given the complex and multifaceted consequences of climate change, including extreme weather, political polarisation, intergenerational impacts, and societal instability [6,16]. The absence of a qualitative understanding of people’s climate-related fears and hopes presents an opportunity to uncover themes that may have been overlooked due to the constraints of quantitative methods (e.g., closed-response items) and to build on the limited qualitative evidence available across different cultures and age groups [18–20], providing valuable content for those aiming to develop fear and hope appeals for interventions (e.g., climate communicators).

By using an inductive approach with open-ended questions, researchers can gain deeper insights into the subjective experiences and perceptions of participants, enhancing our understanding of the phenomenon under investigation [21,22]. Moreover, qualitative methods provide critical insights into the target population, which is essential for designing interventions that utilise fear or hope, whether through emotional appeals, policy design, or community resilience efforts. As Geiger et al. [13] pointed out in a recent meta-analysis of hope-based appeals, there is a need to understand what people are hopeful about to fully understand how hope relates to climate action. Similarly, understanding what people are specifically fearful about can enhance understanding of how fear relates to climate action. Given that different countries, and even segments within a population, may fear or hope for different things regarding climate change, understanding these nuances is particularly important. For instance, while Australia faces a significant risk from more intense and frequent extreme weather events like bushfires, floods and extreme heat [23,24], Pacific Island Nations grapple with rising sea levels that threaten to submerge populated areas [23].

Understanding public perceptions of complex issues like climate change is crucial for effectively communicating risks, motivating behaviour change, designing policies, and informing interventions [25]. For example, in emotional appeals, selecting content that is identified as fear or hope-inducing by the target audience can enhance effectiveness by increasing personal relevance and emotional impact [26,27]. This heightened relevance may, in turn, motivate individuals to change their behaviour to avoid undesirable outcomes out of fear or to achieve desirable ones out of hope [28,29]. Similarly, in policy design, understanding a population’s unique fears can guide policymakers in determining which fears to address or alleviate [30], while understanding their hopes can help in harnessing support for climate policies [9,18]. Regarding community resilience, especially following a natural disaster or extreme weather event, anxiety about climate change, which can lead to adverse mental health outcomes [31], may be mitigated by fostering hope [32]. Drawing on insights from Van der Linden et al., [33] about recommendations for enhancing public engagement with climate change, interventions aimed at alleviating fears about climate change or by fostering hope are likely to be more effective if they are relevant to and reflective of the target population’s specific fears and hopes regarding the potential outcomes of climate change.

### The current study

The current study aimed to explore and understand Australians’ perspectives on what they find fearful and/or hopeful about climate change. We sought to achieve this aim by exploring what content and/or situations induce climate change-related fear or hope in Australians. Particular attention was paid to identifying content and/or situations specific to fear and hope (i.e., uniquely fearful and uniquely hopeful).

## Materials and methods

### Participants

After excluding participants who did not re-consent for their data to be used following the completion of the survey (*n* = 15), the final sample included 299 Australians (*M*_age_ = 33.09, *SD*_age_ = 12.14, age range = 18–81 years; 160 women, 135 men, 2 non-binary, 1 genderfluid, 1 masc), who were recruited and completed the survey on 01/12/2021. This sample size exceeds Braun and Clarke’s [22] recommendation of recruiting more than 100 participants for a large online qualitative study. Qualitative approaches aim to achieve transferability rather than generalisability [22], in that they should provide enough detail to determine how much the current study’s findings can be ‘transferred’ to similarly matched people and/or contexts [34,35].

Participant responses revealed that most participants were predominantly tertiary educated (over 50% held at least one degree) and resided in one of Australia’s three key metropolitan areas (e.g., Queensland, New South Wales, and Victoria). Despite recruitment notices not mentioning climate change or politics, the sample was slightly politically left-leaning, and most identified as environmentalists and believed in anthropogenic climate change (91.3%). Over half of the sample had experienced an extreme weather event (e.g., bushfire, flooding, heat wave) in the last 12 months (throughout 2021) (55.4%).

### Data collection

The study received ethical approval from Deakin University (HEAG-H 184_2021). Participants were recruited via the third-party online recruitment platform Prolific (www.prolific.com) and completed a qualitative online survey via Qualtrics. To prevent discouraging potential participants who were sceptical about climate change from taking our survey, we advertised that the study was investigating Australians’ views on science and social issues. A qualitative survey was chosen over other more interactive modes of qualitative data collection (e.g., interviews). Doing so resulted in a larger corpus, which allowed us to have a more expansive overview of people’s general feelings of fear and hope towards climate change. Using an online survey also allowed participants to provide in-depth responses to a potentially controversial topic (e.g., climate change), increasing the likelihood of providing more genuine responses [22]. A deeper, more nuanced exploration of participants’ perspectives was therefore achieved, resulting in a comprehensive yet standardised summary of the perceptions held by Australians [22,36].

Data collection occurred during December 2021. Participants were eligible to participate if they were 18 years of age or older and resided in Australia. The plain language statement described the study’s purpose, that no identifying information would be collected and the rights of participants in the research. After participants provided informed consent, they then answered two open-ended questions, which were randomised to prevent any order effects (see Table 1). Participants’ socio-demographics (see Table S1 in the S1 File) were collected last to reduce the chance that demographic items would prime participants’ responses to the open-ended questions. We also provided participants the opportunity to give feedback through an open-text response. Given the potential for participants to become upset by asking what they find fearful and hopeful about climate change, contact information for organisations to support them was provided at the end of the survey, along with the option to withdraw their consent now that they had completed the study. Participants were paid £1.15 upon completion of the study.

**Table 1:** Open-ended question schedule.

Emotion	Question
**Fear-related question**	What makes you fearful about climate change?
**Hope-related question**	What makes you hopeful about climate change?

### Data analysis

We employed a participant-driven, inductive approach to data analysis, utilising a contextual epistemology and descriptive theoretical framework during this process [37]. Contextualism notes that there is no single ‘truth’ to be uncovered, but that the data obtained from a study will be valid and ‘true’ for the population and social context it is collected from [21]. Descriptive frameworks focus on providing “a comprehensive summary of events in the everyday terms of those events”, taking participant responses at face value, with little in-depth interpretation occurring [37]. Taken together, this means we developed data-driven conclusions, keeping to the surface level of participant responses and acknowledged that the way participants made sense of climate change depended on their social context. A template thematic analysis was utilised due to its alignment with descriptive, inductive qualitative approaches, as it provides both a structured yet flexible approach to analysis [38]. This includes the development of a codebook after initial data immersion and the allowance to make changes to the codebook and themes through the analytic process.

The template thematic analysis was conducted in the following manner. Step 1 involved reading the corpus to achieve deep immersion and familiarity with the dataset. Both MM and AK read the entire dataset three times, separately, focusing on identifying any meaningful perspectives seen commonly across participants. Step 2 involved the generation of categories into emerging clusters or ‘themes’, with preliminary codes developed and ‘placed’ under relevant themes, resulting in a preliminary codebook or ‘template’. MM and AK held various one-on-one discussions to develop this preliminary codebook, generating ‘working’ themes and relevant codes from their initial impressions and notes made separately in Step 1. Step 3 involved checking the usefulness of the template in answering the research questions. MM and AK proceeded to re-read the dataset once more, separately, and any codes or themes that did not answer the research questions were discarded at this stage. Step 4 involved the iterative modification of the preliminary template. MM conducted a first pass of coding on the entire dataset using the preliminary template. MM and AK then came together to discuss the effectiveness of the preliminary template, removing, moving, adding, or changing themes or codes that did not adequately capture the dataset during this first full pass of coding. Step 5 involved cross-checking the template with alternative views, increasing the credibility of the template. Without seeing the template, EK and KB read the dataset in full (as MM and AK did in Step 1). They then held discussions with MM and AK, providing any additional perspectives that may have been missed in Steps 1 to 3. This resulted in changes to the coding names and template until a finalised template was produced that captured all sections of the corpus relevant to the research questions. Step 6 involved a final coding of the dataset using the finalised template developed in Step 5. MM coded the entire dataset using NVivo. Double coding was then undertaken by AK on 10% of the dataset. A final list of themes was then generated through discussion with MM, AK, and KB, which involved authors reviewing and discussing the coded transcripts and collaboratively working to confirm the final list of themes and subthemes. A reflexivity statement that outlines the researchers’ positionality, sociocultural context, and viewpoints, which contributes to data analysis and interpretation, is presented in the supplementary file.

## Results and discussion

Given that we were interested in the content and/or situations that uniquely induced climate change-related fear and hope, it was decided that participants’ fear and hope responses would be analysed separately. The average word count for fear-related responses was 16 (range = 1–123), while it was 15 for hope-related responses (range = 1–126). Almost all participant responses suggested they were either fearful (*n* = 288), hopeful (*n = *266), or both (*n* = 232). Only a small number of participants reported not feeling fearful (*n* = 11, 3.68%), not feeling hopeful (*n* = 33, 11.04%), or neither feeling fearful nor hopeful (*n* = 6, 2.01%).

Six synthesised themes, three for fear and three for hope, were generated across the dataset (see the supplementary file for detailed descriptions of each theme for fear (Table S2 in S1 File) and hope (Table S3 in S1 File)). No significant changes were made to the quotations presented below. Participant quotes are represented numerically rather than with pseudonyms, given the anonymous nature of the data and the sample size (e.g., P1 = participant 1). To help contextualise participant responses, each quote includes the participant’s age, gender (M = Male; F = Female; NB = Non-binary), geographical region (remote, outer regional, inner regional, metropolitan), and state or territory (Victoria (VIC), New South Wales (NSW), Queensland (QLD), Australian Capital Territory (ACT), Western Australia (WA), South Australia (SA), Tasmania (TAS)). The quotes below are therefore presented in the following format: “*Climate change is scary*” (P1, F, 33, Metropolitan VIC).

While several themes were generated from the data (see 3.1 onwards), the overall corpus did demonstrate three key characteristics across participant responses. The first was the tense used when discussing climate change, where some participants used the present tense to describe their fears and hopes, while others used future tense. The second was the use of value-laden language when describing what they found fearful and hopeful about climate change, including the use of egoistic (i.e., concern for the self), altruistic (i.e., concern for others) and biospheric (i.e., concern for the environment) like statements. The third was how participants’ perspectives on climate change were akin to the idiom “two sides of the same coin”. This metaphor aptly captures that the source of fear and hope was sometimes shared but emphasised differently using a negative or positive frame (see Table S4 in the S1 File for more details).

### Climate-related fear

#### Fear Theme 1: Change and instability.

A dominant perspective across participants was how there was an overwhelmingly rapid rate of (often) negative change and instability that was occurring (or predicted to occur) due to climate change (*“Everything. The prospect that life as we know it is going to change”* P85, F, Metropolitan QLD)*.* That is, participants often indicated they were fearful about climate change because it was significantly changing and destabilising the natural world and their safety within it (*“not knowing how bad things could get and what we do know all sounds terrible and complicated”* P112, F, 29, Metropolitan NSW), with this destabilisation a direct consequence of climate impacts like *“resource insecurity”, “extremely hot temperature”,* or *“rising sea levels”*. This instability was seen to be both current (*“Our way of life is no longer sustainable”* P31, M, 31, Metropolitan VIC) and never-ending (“*What will happen to everything (biotic and abiotic) if we can’t fix what we’ve already broken”* P93, M, 36, Metropolitan NSW), which only increased participants sense of foreboding and fear (*“The thought that the world could radically change in the coming years and may be uninhabitable for humans”* P95, M, 21, Metropolitan QLD).

When participants discussed their fear of the growing instability due to climate change, certain contexts in which these rapid and negative changes were seen to occur were often mentioned. These included *“changes in our way of life”* (P118, M, 19, Metropolitan SA)*, “natural landmarks like the Great Barrier Reef*” (P80, F, 20, Metropolitan NSW), *“people having to relocate their homes”* (P87, M, 62, Metropolitan WA)*, “disruption of food and water supplies”* (P23, M, 50, Inner Regional TAS) and *“damage to forests and other habitats for animals and people”* (P26, F, 57, Metropolitan SA)*.* Overwhelmingly, however, were mentions of changes in the natural environment as being fear-inducing *(“The unknowns of when and if extreme weather will cause more natural disasters”* P18, F, 39, Metropolitan VIC*).* Here participants mentioned specific and *“unpredictable”* weather events (*“Catastrophic weather events (e.g., once in a decade floods, massive bushfires)”* P66, M, 28, Metropolitan QLD), an increase in the frequency of natural disasters (*“Natural disasters happening more often”* P73, F, 39, Metropolitan NSW), and overall changes in the natural climate (*“Extremely hot temperatures and no cooling”* P76, M, 44, Outer Regional SA)*.*

The idea of *“survival”* for humans, animals and nature was also a consistent response, with several participants noting they feared how climate change could lead to parts of the world becoming uninhabitable (*“The thought that the world could radically change in the coming years and may be uninhabitable for humans”* P95, M, 21, Metropolitan QLD)*.* Some were concerned about the habitability of animals (*“Animals losing their habitats”* P170, M, 22, Metropolitan VIC), while others referred to natural habitats more generally (*“Degradation of natural habitats”* P281, F, 25, Metropolitan NSW)*.* Yet one response succinctly captured participants’ fear of survival simply stating: *“It is change; change is not stable; instability leads to death”* (P43, M, 33, Inner Regional QLD).

Beyond the changes in the natural environment, participants also noted they were fearful of the wider alterations climate change would have on them and their way of life. At times this was discussed in terms of *“changes in the way the world operates”,* with specific reference to cost-of-living pressures (*“Not being able to afford standard of living”* P113, F, 22, Metropolitan VIC), while others were fearful of how their health and wellbeing would be impacted. This included being fearful of the increasing risk of *“physical”* and *“mental”* health problems and the increase in *“diseases”*, as well as overall *“death”* and *“human extinction”*. One participant who worked in healthcare even noted how they were fearful of “*people dying”* due to *“smoke or heat stroke”* and that this fear was intensified because *“it is avoidable and tragic*” (P254, F, 26, Metropolitan VIC)*.* However, some referred to the health impacts on themselves and others in more abstract terms (*“Understanding that I will be less able to protect myself and loved ones, including young children, from the worst of climate change (e.g., bushfires)”* P269, F, 31, Metropolitan VIC). This lack of clarity appears to align with the uncertainty people experience regarding how the effects of climate change will materialise, as its impacts are far-reaching and challenging to comprehend [4].

When discussing the type of climate change instability they feared, participants also referred to the consequences these rapid changes would have for society. This included *“population displacement and forced migration”* (P255, M, 41, Inner Regional VIC)*, “global conflicts”* (P208, M, 38, Metropolitan NSW) and *“collapse of modern society”* (P214, M, 30, Metropolitan NSW)*.* For example, some participants noted that climate change could lead to social unrest (*“I am scared of civil unrest and potential totalitarianism in response”* P129, F, 31, Metropolitan VIC), suggesting that some people were fearful that the potential outcomes of climate change, such as environmental destruction and competition over resources, may result in domestic and international conflict. Other participants discussed the potential of resource insecurity, especially food and water shortages, as something to fear (*“Disruption of food and water supplies. Also, risk of war arising from those shortages”* P23, M, 50, Inner Regional TAS)*.* Other responses acknowledged the inequality of climate change impacts, with participants mentioning climate migration and population displacements were also something to be fearful of (*“All the climate refugees that will lose their homes”* P222, M, 26, Metropolitan VIC).

#### Fear Theme 2: Inaction and negligence by government, corporations, and others.

Another climate change related fear participants noted came from the systems and authorities responsible for causing climate change, and their unwillingness to mitigate it. This included such authorities and systems as: *“commercialisation”, “capitalism”, “the coal mining sector”, “the inaction of governments”, “the political divide and misinformation”, “corporate greed, “people in power”, “corruption”* and *“apathy”.* Additionally, similar fears were expressed towards the general population that either denied the existence of climate change (“*some people still deny it exists”* P100, M, 18, Metropolitan NSW) or who did not care enough to alter their behaviour (*“people’s lack of care for the impact their own actions have”* P115, F, 19, Metropolitan QLD)*.* Therefore, it appeared that participants not only feared the change and instability from climate change (see Theme 1 above), but they also feared the human barriers (e.g., power, greed, money) of significant, systemic inaction, especially from governments and major corporations. This perception aligns with recent research indicating that young Australians (15–19 years) are worried about how their futures are dependent on decisions of current leaders [39]. Specifically, this *“lack of seriousness and urgency”* (P130, F, 30, Metropolitan VIC), and instead the greed and/or apathy of these organisations, was seen as fear-inducing as it limited the global community’s ability to address climate change. As succinctly stated by some participants:

*“What makes me truly fearful isn’t climate change because I believe humans have the capacity to innovate out solutions. However, with the greed apparent in the upper classes and the corruption in the government with Morrison [Scott Morrison, Prime Minister of Australia at the time the data was collected] at the helm makes me fearful”* (P102, M, 21, Metropolitan VIC).

*“The mega-rich wasting money going into space. Governments and big business who don’t care, govts. only care about power and votes and businesses only care about money and* *optics”* (P39, F, 47, Metropolitan NSW).

When examining this theme more closely, many participants referred to the lack of government action or to governments’ negative influence in perpetuating climate change, a sentiment consistent with expert assessments at the time [40]. For example, while some participants consistently mentioned how the *“Australian Federal Government”* and their *“slow rate”* and/or resistance to mitigating climate change was fear-inducing (*“Australia’s lackadaisical policies on doing anything”* P177, Masc, 22, Metropolitan NSW), others expressed fears about the entanglement between government and business (*“The Australian government works for the fossil fuel industry”* P111, F, 59, Metropolitan SA) as well as politicians vested interests in maintaining *“power”* (*“Lack of foresight from most governments, too much focus on re-election rather than solving problems”* P191, M, 42, Metropolitan NSW). In contrast, other participants explicitly noted they were fearful of large corporations, including their contribution to climate change and their resistance to meaningful climate action (*“I am fearful about climate change because I do not see the needs and wants of big business being put on the back burner in the face of impending doom”* P282, F, 19, Metropolitan QLD), noting how corporations had vested interests (for example, investments in *“traditional power generation sources”* P74, M, 43, Metropolitan QLD) in not taking climate action (*“big corporations and governments that invest in short cuts that are bad for the environment but make them money”* P119, F, 20, Metropolitan VIC). Nevertheless, most participants were fearful that the current socio-economic system did not facilitate or incentivise either governments or big businesses to take necessary actions to significantly mitigate climate change, as clearly noted by one participant:

*“The fact that commercialisation & industry & capitalism means that it is only going to get worse as governments and big businesses care more about making money than being environmentally conscious”* P4, M, 27, Metropolitan NSW.

While some participants discussed how they feared the lack of action and negligence by the government and large organisations, others expressed that they feared disengagement and lack of concern shown by others in the general population. Here, participants explicitly flagged *“climate change deniers”*, *“humanity”*, and *“selfishness”* as all things they currently feared. Some participants even mentioned how the *“media”* and *“news reports”* represented climate change was something that made them fearful (“*Seeing news reports about future projections of climate change. Seeing images of flooding, fires, hurricanes”* P65, M, 23, Metropolitan NSW).

Interestingly, some participants also reported that individuals who denied the existence of climate change or did not acknowledge their behaviour’s contribution to the problem, was fear-inducing. For example, when explicitly referring to climate change deniers, participants noted how they feared the way they blocked progress (*“That it requires a collective effort to curb it, which seems like a massive challenge given the climate change deniers”* P67, F, 29, Metropolitan VIC) and continued to debate the existence of climate change (*“Disagreements about its existence”* P118, M, 19, Metropolitan SA)*.* In contrast, others said that they were fearful of the little care shown by others (*“People don’t seem to care about all the resources they use and the pollution that is created that is damaging the environment”* P26, F, 57, Metropolitan SA), with one participant pointing to *“self-interest grou*ps” and *“religiosity”* specifically (P98, M, 75, Metropolitan QLD). Despite an increase in the number of Australians alarmed about climate change [41], participants within this theme appeared to continue to grapple with the pervasive fear of climate change denial and carelessness shown by others.

Along similar lines, participants’ responses also described how they were fearful and doubtful that humanity would be able to tackle climate change meaningfully. For example, one participant demonstrated this by stating that *“Humans are incapable of changing”* (P14, M, 32, Metropolitan VIC). In contrast, others expressed their dissatisfaction with humanity’s current efforts in mitigating climate change by using the possessive determiner *“we”* to signify their evaluation of collective efforts (*“we are not doing enough”* P160, F, 28, Metropolitan VIC) and the slow rate of this change (e.g., *“That we aren’t making changes fast enough” P169, F, 30, Metropolitan NSW*). These responses indicate potentially deep-seated fears and doubts about humanity’s capacity to effectively *“deal”* with the challenges of climate change. The added layer of urgency indicated by the belief that *“we”* may not be able to halt the trajectory of climate change suggests that people’s opinion is that accelerated and collective action may be needed to address climate change effectively.

#### Fear Theme 3: Intergenerational impacts and legacy.

This final theme captures how participants also discussed how fearful they were for future generations due to climate inaction, specifically demonstrating angst about the *“future”* and young people’s experience of the *“planet”* in the not-so-distant future *(“I am fearful as it will make the planet a terrible place to live in the future”* P199, M, 51, Metropolitan WA). This included a general fear for *“younger generations”* and *“future generations”* but also a fear for their current or future *“children”*. Some younger participants even noted how they were fearful for their future. As one participant aptly noted:

*“I’m fearful that as a young person I will be alive to see negative changes to the earth and possibly see the earth become uninhabitable. I’m also worried about the state of the earth for future generations, especially if I have kids of my own one day” P206, F, 19, Metropolitan VIC).*

Many of the participant responses in this theme were connected to *“children”,* with participants often using the possessive determiner such as *“my”* and *“our”.* For example, some expressed concerns about the quality of life their children might endure because of climate change (*“That we will make the planet unliveable for my children”* P137, M, 46, Metropolitan WA). Others referred to the idea of what current generations would leave behind for young people to inherit (*“leaving our planet in a much worse state for future generations”* P91, F, 29, Metropolitan QLD). The use of possessive determiners and the idea of leaving a degraded planet for future generations suggests that many participants experienced a sense of responsibility, as well as fear, for how climate change would impact younger people in the future.

This theme is consistent with other qualitative research investigating parents’ perceptions of climate change, indicating that people are concerned about how the adverse effects predicted by IPCC [1] are likely to impact their children’s lives [42]. In particular, parents expressed sadness, hopelessness, and anxiety when considering how climate change will impact their children, specifically referring to environmental degradation, species extinction, and quality of life [42]. Similarly, when asked, *“How do you feel about climate change?”* Australian scientists also revealed concern for current and future generations, explicitly referring to their own children. Engaging in climate action to protect current and future generations was found to be a top priority among North American participants [43]. The current study’s findings build upon this research by showing that Australians fear how climate change will impact current and future generations.

### Climate-related Hope

#### Hope Theme 1: Changing attitudes and changing pro-environmental habits.

While in Fear Theme 2 (see above) participants expressed a fear of sceptical climate change attitudes and behavioural disengagement in the issue, many participants also described that people becoming aware of climate change, and their individual impact on the issue, made them hopeful for the future. This was especially evident when participants said they felt hopeful when seeing people who *“care”,* who “*are listening”*, and who are *“changing their ways”*. Much of the language utilised in this theme also aligned with dynamic norms (i.e., the positive shift in pro-environmental engagement overtime among society) [44] (*“Human behaviour changing & making more of an effort to reduce carbon footprint & take more sustainable approaches”* P236, F, 40, Metropolitan VIC).

For most participants, a frequent source of hope was the public’s awareness of climate change, with participants using terms such as *“accepting”, “care”, “attention”, “interested”, “listening”, and “engagement”* to demonstrate this view*.* Some even emphasised that the ongoing and active processes of “*increasing awareness”* was hope-inducing, using such phrasing as “*becoming aware”, “growing awareness”,* and *“more people beginning to care”*, thereby showing the dynamic nature of hope of participants. Moreover, this source of hope appeared to come from the observation that more people in society had shifted from climate change scepticism to *“acceptance”* and were taking it more *“seriously”* (e.g., *“People are starting to accept its happening*” P6, F, 25, Metropolitan VIC). A few participants even noted how *“schools”* that were now educating students about climate change, thus increasing awareness among young people, made them feel hopeful (*“Schools are teaching students about greenhouse gasses and some things that need to be changed in order to be on track to rid climate change”* P9, M, 24, Metropolitan NSW).

Another source of hope for participants was their observation that more individuals were *“making a change”* and engaging in individual pro-environmental behaviours. This was reflected in such phrases as: *“people are making small changes”, “individual change”,* and *“actions of individuals”*. While some participants’ responses described how norms were changing (*“More and more individuals are changing their ways”* P73, F, 39, Metropolitan NSW), others specified behaviours such as *“reducing plastic waste”, “favouring electric cars”, “reducing fast fashion”,* and *“changes to their diet”* as hope-inducing. These perspectives align with previous research on American adults and Swedish teenagers that found witnessing others engage in pro-environmental behaviours was a source of hope [18–20], and that individuals respond positively to depictions of ordinary people engaging in pro-environmental behaviour [45]. Our study extends these findings by showing that Australians also find hope in seeing ordinary people engage in pro-environmental behaviour.

Participants also noted how collective efforts to combat climate change made them hopeful, frequently using terms like *“community”, “public”,* and *“people”* (*“Collaborative global efforts to affect change and/or prevent further damage” P209, 33, F, Metropolitan NSW).* Some even emphasised how *“many people”* and *“countries”* were working together to address climate change inspired hope (*“That there are people working to make a change and that there are a lot of people that support this”* P67, F, 29, Metropolitan VIC). Some even noted how *“humanity’s”* capacity to confront *“challenges”* in the past raised their hopes in tackling climate change, pointing to the human characteristics of *“resilience and resourcefulness”* and *“intelligence”* as essential to this endeavour*.* From this perspective, participants’ hopes were closely intertwined with their belief in collective action and humanity’s resilience. These insights shed light on the multifaceted nature of hope in the context of climate change, emphasising the significance of community involvement and the perceived innate human ability to address environmental challenges when there is the desire to do so.

Interestingly, while in Fear Theme 2 (see above) expressed fear towards the inaction of governments and businesses, it appeared they experienced hope when thinking about collective community driven movements for climate action. In fact, for some participants, the resurgence in climate activism and how it could force governments and large corporations to *“change their ways”* also induced hope, indicating that seeing or knowing others engage in activist behaviour (not just individual behaviour) was something that made them hopeful *(“there seems to be a growing movement that are recognising the dangers and campaigning for change”* P252, M, 47, Metropolitan QLD). One participant went so far as to say that they found hope in seeing others engage in digital activism*:*

*“That there are people who are trying to mitigate it by picking up trash from the ocean, planting trees and are using their social media platforms to influence other people to join in”* (P241, F, 19, Metropolitan NSW).

Similarly, in the context of *“increasing pressure on decision-makers”* (P129, F, 31, Metropolitan VIC), another participant specifically pointed to climate change protests that occurred in Melbourne and Sydney in 2019*:*

*“The climate change rallies/protests in Melbourne and Sydney in 2019 show that this is an issue that more people are becoming concerned about, which may pressure the Australian Government to do more to address climate change”* P67, F, 29, Metropolitan VIC)

While prior research has found people respond negatively to environmental activism, especially radical activism [46], participants in the current study instead tied some of their hope to the burgeoning wave of climate activism, where individuals and groups, through their campaigns, protests, and digital endeavours, actively engaged in advocating for environmental action. This collective push, epitomised by events like the climate rallies in Australian cities, may reflect a growing societal commitment to addressing climate change and the hope that collective action can be successful. Although in Fear Theme 3 (see above) many participants felt fearful for the future of young people, several were also hopeful due to younger generations being the most *“fired up”* demographic in mitigating climate change. When discussing this youth engagement, participants often referred to activism or activist-like behaviours carried out by young people as being hope-inducing *(“Young activists taking a stand for change”* P201, F, 20, Inner Regional SA), with one participant aptly noting that they were hopeful because “*slowly younger people are making their voices heard”* (P45, F, 28, Metropolitan VIC). One participant even mentioned the School Strikes for Climate, also known as Fridays for Future, noting that seeing these events made them feel hopeful that progress was possible because of *“Young people like Greta Thunberg and everyone else who is campaigning for action to be taken now”* (P39, F, 47, Metropolitan NSW).

Overall, viewing others’ behaviours as hope-inducing is somewhat unsurprising given that prior research has shown that people observing others engage in climate activism can increase collective efficacy beliefs (i.e., humanity’s ability to mitigate climate change effectively) [47]. Further still, other findings indicate that familiarity with Greta Thunberg, the pioneer of school strikes for climate and often depicted as the symbol of youth climate activism, is positively associated with collective action intentions and collective efficacy beliefs [48]. In the current study, responses about young people and climate activism suggest that observing or being aware of young people’s engagement with the issue of climate action could trigger feelings of hope.

#### Hope Theme 2: Progress, sustainability, technology, and innovation.

While Fear Theme 2 (see above) emphasised how governmental and industry inaction resulted in fear of participants, this theme reflects how participants felt hope when scientists, governments, and corporations were seen to be meaningfully working towards change. This was reflected in responses that pertained to participants hopes about *“progress”* in mitigating climate change and advancements in *“technology”, “initial actions towards sustainability”* (P173, M, 39, Metropolitan QLD)*, and “scientists…working hard”* (P186, F, 31, Metropolitan ACT). For instance, some emphasised the future promise of science and technological solutions, specifically noting that their hope arose from the potential of *“technological advances”* and *“innovation from scientists”*. Others noted that their hope was that action on climate change had the *“potential for a surge in development of better technologies”* (P23, M, 50, Inner Regional TAS), and that scientific and technological solutions would be found that did not require “*people to change their habits”* (P1, F, 35, Inner Regional VIC) (*“Surely there must be some scientific and technological solutions which will eventually be brought to bear on the issue”* P166, M, 47, Metropolitan QLD). A small number of participants even pointed to historical actions from governments and businesses to mitigate the effects of human behaviour on the environment as a reason to be hopeful (e.g., *“In the 90’s there was a lot of emphasis on the hole in the ozone layer, which led to reforms in CFC usage/refrigerants etc. that ultimately addressed the issue”* P288, F, 38, Metropolitan NSW).

Within this theme, participants also reflected feeling hopeful about progress or advances in specific forms of technology, primarily renewable energy. Multiple participants made explicit reference to *“renewable energy”,* with some even saying they were hopeful because Australia was a prime location for renewable energy to be successful (*“Renewable Energy and Australia is abundant in it”* P49, M, 31, Metropolitan VIC)*.* Some even pointed to the possible positive economic outcomes of adopting renewable energy and environmentally friendly alternatives, noting it will result in *“more jobs”*, that *“Green hydrogen will make manufacturing cheaper in Australia”* and that *“Australia has the potential to export green energy”* (P111, F, 59, Metropolitan SA).

Participants’ belief that technology and science alone are sufficient to mitigate climate change has also been found in prior research. For instance, holding strong beliefs that technology and innovation will solve climate change appears to diffuse individual responsibility for mitigating climate change, decrease risk perceptions, and increase behavioural disengagement [19,47]. Furthermore, relying on external sources (government intervention, technological advancements) in solving complex issues, such as climate change, is associated with a personal lack of control [12,49]. When this occurs, a reduction in motivation to confront and mitigate climate change is more likely due to a reduction in urgency and an increase in complacency [12,50]. While technology and science appear to increase climate-related hope in participants, this prior research also suggests that inducing hope by describing progress in government interventions and technological advancements may also result in counterproductive attitudes, such as relying solely on technology.

#### Hope Theme 3: An opportunity for change.

While less commonly seen across participants, Hope Theme 3 outlines how some participants saw the potential benefits of climate change as a source of hope, suggesting that participants were engaging in a positive reappraisal of climate change itself (“*Positive changes to the human experience if we deal with it correctly”* P109, F, 27, Metropolitan NSW). For instance, some indicated that they hoped climate change would catalyse action, which was reflected in participants using words such as *“make”, “drive”,* and *“force”* when talking about the positive changes that could arise from climate action. Similarly, participants also expressed that they were hopeful that “*feeling the effects of climate change”*, such as experiencing extreme weather events (see Fear Theme 1 above), would be a *“wake-up call”* for people and governments to “*do something about it”*. As one participant noted: “*Humans in general aren’t great at dealing with problems proactively so hopefully with enough unavoidable* *problems there will be incentive to act”* (P288, F, 38, Metropolitan NSW).

While some participants engaged in a positive reappraisal of climate change, this reappraisal did differ across participants. Some referred to more human-centred opportunities for innovation and positive changes to the human experience (*“I think that there are opportunities for innovation and positive changes in the way we live, e.g., more solar panels, less carbon footprint”* P121, F, 26, Inner Regional VIC). In contrast, others mentioned the potential environmental benefits of climate change for *“species”, “flora”, “fauna”,* and the *“planet”*, noting this could result in positive adaptations or opportunities for certain species (*“Some species may actually benefit from climate change and others might adapt to it”* P32, M, 32, Metropolitan VIC).

Overall, it appeared that for some participants, there was hope that the negative impacts of climate change could catalyse public pro-environmental change to avoid future climatic disasters, a phenomenon recently referred to as ‘transilience’. Transilience contains three components that capture how people adapt to climate change, one of which is the ability to transform positively in response to climate change [51]. However, transilience refers to the individual benefits of responding to climate change, whereas most responses in this theme refer to collective benefits. Nevertheless, significant societal changes can be triggered by opportunities to address the underlying causes of disasters [52,53]. For instance, the widespread adoption of low-carbon alternatives from traditional power sources in response to extreme weather events or natural disasters linked to climate change. While positive reappraisals have been linked to increases in pro-environmental behaviours in adolescent populations (< 18 years) [32,54–56], this theme suggests that Australian adults may also engage in positive reappraisal, emphasising the opportunities to cope with climate change effectively.

### Implications and future directions

Climate change communicators (e.g., government agencies and NGOs) can leverage the quotes and insights from participants to better engage their target audience (especially Australians) through emotional appeals or other communication strategies. For instance, the prominent fear theme of ‘Change and Instability’ provides specific content that communicators could use to highlight the potential consequences of unmitigated climate change, such as threats to the habitability of Earth for humans, animals, and plants. However, audience members may be more likely to change their behaviour if these consequences align with their value orientations. For instance, individuals with egoistic values may respond better to threats that align with the risk of personal harm (e.g., from exposure to extreme weather events), where those with biospheric values may respond better to environmental threats (e.g., biodiversity loss) [57]. Conversely, the hope theme of ‘Changing Attitudes and Changing Pro-environmental Habits’ illustrates the potential of emphasising dynamic norms (i.e., highlighting positive changes in society attitudes and behaviours over time) to induce hope [18,44]. Future work should experimentally test the effectiveness of messages that incorporate insights from these themes. Additionally, using further qualitative methods, such as interviews and focus groups, can help explore what specifically makes certain stimuli fear or hope-inducing [22].

While this study focused on exploring the climate-related content that uniquely made Australians fearful or hopeful, some of the generated themes aligned with similar studies conducted in other countries. For example, the fear theme ‘Intergenerational Impacts and Legacy’ aligns with quantitative findings from Mullins-Jaime & Watcher [43], who found that American and Canadian participants motivated to mitigate climate change were driven by the desire to protect current and future generations from the impacts of climate change. Additionally, the hope theme ‘Changing Attitudes and Changing Pro-environmental Habits’ aligns with studies indicating that rising awareness of climate change severity and witnessing others engaging in pro-environmental behaviour were common reasons for hope among American adults [19] and Swedish teenagers [20]. These similarities suggest that certain content may induce climate-related fear and hope across cultures. However, given these studies, including the current one, used samples from the Global North, future research should seek to investigate what participants from the Global South (e.g., Indonesia) find fearful and hopeful about climate change and whether the similarities mentioned above are still evident. Understanding what fear and hope-related content may be relevant across Global North and Global South nations could further clarify which themes are universally relevant and which are nation-specific.

The generation of ‘An opportunity for change’ as a unique theme provides valuable evidence about how participants conceptualise hope amid the current climate crisis (e.g., rising temperatures, extreme weather events, and biodiversity loss). This theme reflects a form of utopian thinking (i.e., positive visions for the future of society [58]), where participants envision transformative societal, environmental and economic changes in response to worsening climate conditions. Such thinking is associated with societal engagement in utopianism research [58], suggesting this kind of thinking may help drive change motivation. Additionally, research has demonstrated that a ‘green utopian vision’ (i.e., a future based on sustainability and efficiency) may be particularly motivating [59] and relatively prevalent in the population [60]. Furthermore, recent research has found that positive future utopian visions can invoke hope and indirectly increase collective climate action and mitigation intentions via the induction of hope [61,62]. However, given the limited qualitative evidence available related to the content of people’s utopian visions in climate change [58], understanding what people are hopeful about can help create communication strategies that adopt positive utopian thinking. For instance, it could be effective for climate communicators to frame climate change as an opportunity to reimagine what society, the environment, and/or the economy could be like in the future, gaining support for system-changing policies to achieve the target of net zero emissions by 2050.

### Limitations

Although the current study provides several benefits to the climate communications literature, the study’s limitations must be noted. Firstly, data collection for this study occurred in December 2021, four months before the Australian Federal election, in which climate change was a prominent issue. At this time, many Australians were unhappy with the level of climate action the Australian Liberal National Party had engaged in during their political term (from 2013 to 2022) [63]. Unsurprisingly, the negative sentiment toward the federal government was frequently seen in participants’ responses to the fear and hope-related questions. However, while the Australian Labour Party (ALP) took office on 1st May 2022, it is still likely that Australians would continue to identify the lack of governmental action as a source of fear, given the continued approval of new coal mining projects since May 2022 [64].

Beyond this, most of those who participated in the study lived in metropolitan Australia (See Table S1 in S1 File), and were politically left-leaning, which may have impacted the responses received. For instance, residents of rural Australia are more likely to directly experience natural disasters [65], exposing them to more economic consequences since many of them work in climate-sensitive industries (e.g., agriculture), relative to metropolitan communities [66]. Although metropolitan participants frequently mentioned natural disasters and extreme weather events in their responses, including more rural and regional participants may have added further insightful perspectives, such as the ongoing impacts in the aftermath of natural disasters and extreme weather events (e.g., forced migration) [67]. Regarding political ideology, people who identify as right-leaning are more likely to be concerned with the economic impacts of climate change (e.g., restriction of market freedoms) [68]. Thus, we may have received more responses of fear and hope in the context of the economic effects of climate change if the sample included more right-leaning participants than left-leaning participants. Nonetheless, given the limited diversity in the sample and, therefore, the transferability of the study’s findings, researchers or practitioners (e.g., climate change communicators) interested in applying the study’s results must consider the context in which they intend to use them.

## Conclusion

The current study utilised a qualitative approach to explore and understand Australians’ perspectives on what they found fearful and/or hopeful about climate change. The identified themes outline specific content Australians view as fear and hope-inducing. As expected, the existential threats posed by natural disasters and extreme weather events induced fear, yet inaction by individuals, governments, large corporations, or the intergenerational impacts of climate change were also described as fear-inducing. On the other hand, positive changes in pro-environmental attitudes and behaviour reflecting changing norms, as well as youth movements advocating for climate mitigation, were widely reported as hope-inducing. These findings are particularly relevant to individuals such as communicators, policymakers, and researchers who intend to use fear or hope as a motivator and wish to include emotional content in environmental communications that more accurately aligns with Australians’ fears and hopes about climate change. Seeking to identify fear and hope stimuli unique to the audience may lead to environmental communications that are more emotionally impactful, and subsequently, motivating to engage in climate action.

## Supporting information

S1 File Supplementary materials. (DOCX)
