# Supplementary material for: Understanding what Australians find fearful and hopeful about climate change through qualitative approaches
Source: PLoS One. 2026 Jan 7;21(1):e0339306. doi: 10.1371/journal.pone.0339306 (PMC12779138; doi:10.1371/journal.pone.0339306)
Supplement: S1 File — (DOCX) [file pone.0339306.s001.docx]

**Supplementary Materials**

### **Reflexivity Statement**

Engaging in reflexivity is a vital component of qualitative research, as it ensures that the researchers acknowledge how their positionality, sociocultural context and viewpoints contribute to data interpretation and analysis (Berger, 2015). All authors reside in Australia, experiencing the same impacts of climate change on the Australian landscape as the participants, resulting in primarily insider perspectives used to understand the data. The research team also contained expertise across several psychology disciplines, including social [AK, MM, JF], environmental [AK, MM, JF], health [EK, ML] and personality [EK, ML, KB], as well as qualitative methodology [AK]. This ensured the team was able to identify a wide range of psychological processes within the data. In the context of the current study, all authors have experienced, or know someone who had experienced, an extreme weather event (e.g., heat-wave, cold-wave, thunderstorms, bushfires). All authors are of generations (e.g., millennial) which increase the likelihood that they will experience more of the negative and catastrophic effects of climate change than older generations. All authors research in the area of climate change interventions, and all agree that current efforts to transition from fossil fuels to renewable energy (e.g., solar and wind energy) is a source of hope in Australia. It is important to note that all authors live in urban areas (Melbourne); areas that are less susceptible to climate change which could potentially bias the interpretation of participants responses.

**Table S1**

*Demographic characteristics of participants*

|  | Overall |
| --- | --- |
| *n* | 299 |
| Highest level of education (%) |  |
| Lower secondary education (less than high school) | 3 (1.0) |
| Upper secondary education (high school completion) | 56 (18.7) |
| Post-secondary non-tertiary education (e.g., Technical College, Certificate I to IV, Diploma) | 47 (15.7) |
| Bachelor’s Degree (e.g., BA, BS) | 134 (44.8) |
| Professional or Master’s degree (e.g., MA, MS, MEng, MBA, MD, DDS, LLB) | 46 (15.4) |
| Doctorate (i.e., PhD) | 13 (4.3) |
| Age in years (mean (SD)) | 33.09 (12.14) |
| Gender (%) |  |
| gender fluid | 1 (0.3) |
| man | 135 (45.2) |
| masc | 1 (0.3) |
| non-binary | 2 (0.7) |
| woman | 160 (53.5) |
| State (%) |  |
| Australian Capital Territory | 7 (2.3) |
| New South Wales | 89 (29.9) |
| Queensland | 51 (17.1) |
| South Australia | 27 (9.1) |
| Tasmania | 8 (2.7) |
| Victoria | 93 (31.2) |
| Western Australia | 23 (7.7) |
| Area (%) |  |
| Metropolitan | 254 (85.0) |
| Inner Regional | 33 (11.0) |
| Outer Regional | 11 (3.7) |
| Remote | 1 (0.3) |
| Political ideology^[[1]](#footnote-1)^ (mean (SD)) | 3.52 (2.17) |
| Environmental Identity (mean (SD)) | 6.59 (2.53) |
| Climate change beliefs (%) |  |
| I don’t think climate change is happening | 6 (2.0) |
| I have no idea whether climate change is happening or not | 3 (1.0) |
| I think that climate change is happening | 17 (5.7) |
| I think that climate change is happening, and I think that humans are largely causing it | 273 (91.3) |
| Extreme Weather = Yes (%) | 165 (55.4) |
| Drought = Yes (%) | 15 (5.0) |
| Flooding = Yes (%) | 57 (19.1) |
| Heat wave = Yes (%) | 89 (29.8) |
| Cold wave = Yes (%) | 36 (12.0) |
| Cyclone = Yes (%) | 7 (2.3) |
| Bushfire = Yes (%) | 55 (18.4) |
| Thunderstorm = Yes (%) | 152 (50.8) |
| No extreme weather event = Yes (%) | 91 (30.4) |
| Other = Yes (%) | 9 (3.0) |

**Table S2**

*What makes you fearful about climate change?*

| **Themes** | Definition | Exemplar Quotes |
| --- | --- | --- |
| **Change and Instability** | Participants would often describe outcomes of climate change that destabilised the natural environment or could cause changes that result in environmental destruction or vulnerabilities to the effects of climate change.  This was demonstrated explicitly via participants' discussions of extreme weather and natural disasters, societal and human impacts (e.g., social conflict, physical and mental health), environmental degradation, and threatened flora and fauna. | *“I am also more fearful of increasing severe weather conditions such as the bushfires that Australia experienced in the summer of 2019, when a large majority of the country was on fire” (P64, F, 32, Metropolitan QLD)* |
| **Inaction and Negligence by Government, Large Corporations, and Others** | This theme reflects how participants would emphasise how they feared inaction by individuals, professionals, governments, politicians, or organisations to mitigate the effects of climate change.  Responses in this theme often inferred who is responsible for taking action or inaction and what is responsible for contributing to climate change. This was demonstrated explicitly via participants' discussion of climate inertia and negligence, corruption and vested interests, renewable energy, significant sources of greenhouse gas emissions, and research and scientists. | *“The lack of seriousness and urgency that the federal government and some state governments are displaying with current actions such as new coal and gas projects and terrible behaviour at COP26” (P130, F, 30, Metropolitan VIC)* |
| **Intergenerational Impacts and Legacy** | Participants would describe the potential adverse impacts that climate change was predicted to have on current and future generations.  This was demonstrated explicitly via participants' discussions of familial impacts, generational impacts and legacy. | *“How liveable the world will be for my children and their children.” (P246, F, 33, Metropolitan VIC)* |

**Table S3**

What makes you hopeful about climate change?

| **Theme** | **Definition** | **Exemplar Quotes** |
| --- | --- | --- |
| **Theme 1: Changing Attitudes and Changing Pro-environmental Habits** | This theme reflects participants descriptions of how engagement with the issue of climate change and mitigating climate change shown by others was something they found hope-inducing.  This was demonstrated explicitly via participants' discussions of climate awareness and acceptance, activism and campaigns, climate education, promotion of climate literacy, public pressure on governments and large corporations, behaviour change and eco-preservation. | *“More and more people seem to be understanding how deadly serious this is and what’s needed”* (P130, F, 30, Metropolitan VIC) |
| **Theme 2: Progress, technology, sustainability, and Innovation** | Participants often described how large-scale actions taken to mitigate climate change were a factor in making them feel hopeful about climate change.  This was demonstrated explicitly via participants discussion of restoration and reversible damage, collective climate action, government intervention, the human condition, corporate change, technology and innovation, and alternative energy and clean burning. | *“The amount of technological innovations being made to address climate change. The green tech industry is booming, and the methods we are developing provide me with hope for a better future”* (P152, M, 23, Inner Regional TAS) |
| **Theme 3: An Opportunity for Change** | This third theme aimed to capture participants responses which referred to the benefits and positive outcomes of climate change.  This was demonstrated explicitly via participants discussion of the positive changes to the human experience (e.g., healthy lifestyle changes). It also included responses that explained that they hoped the negative impacts of climate change would lead people to have no choice other than to engage in more pro-environmental behaviours. | *“That it can drive people to make sustainable choices and change habits to reduce activities that contribute to climate change”* (P140, F, 25, Metropolitan SA) |

**Table S4**

*Exemplar quotes of the three key characteristics of the overall corpus.*

| Characteristic | Exemplar Quotes |
| --- | --- |
| Present and future Tense | "That I can already see change happening even though I'm not that old. I can feel the summer being hotter, I can see the extreme weather events happening." P8, M, 27, Metropolitan QLD (present fear)  "Young people are concerned about climate change and they have made many contributions to prevent global warming" P57, M, 29, Metropolitan WA (present hope)  "That there will be a shortage of resources in the future, including water and food shortages" P281, F, 25, Metropolitan NSW (future fear)  *“I am hopeful that things will change quickly soon” P186, F, 31, Metropolitan ACT* (future hope) |
| Value-laden language | *"Not being able to afford standard of living" P113, Female, 22, Metropolitan VIC* (egoistic fear)  "*I am hopeful when I hear that scientists are looking for ways to help changes in the climate that don't require people to change their habits" P1, F, 35, Inner Regional VIC* (egoistic hope)  *"all the climate refugees that will lose their homes" P222, Male, 26, Metropolitan VIC* (altruistic fear)  *"The coming together of community to support ourselves as we are starting to"* *P254, F, Metropolitan VIC* (altruistic hope)  *"Endangered species, destroyed environments, pollution" P155, Female, 27, Metropolitan SA* (biospheric fear)  *"The mandates and initiatives to try decrease climate change and help the environment prosper." P115, Female, 19, Metropolitan QLD* (biospheric hope) |
| Two sides of the same coin | *“That we are leaving a world in a terrible state for our kids and future generations” P68, M, 36, Metropolitan QLD (fear)*  *“The younger generation is very passionate, educated and motivated in regards to climate change. This is the next generation that will be leaders” P168, F, 30, Metropolitan NSW (Hope)* |

**References**

Berger, R. (2015). Now I see it, now I don’t: Researcher’s position and reflexivity in qualitative research. *Qualitative Research*, *15*(2), 219–234. <https://doi.org/10.1177/1468794112468475>

1. Political ideology was measured on a 10-point-sliding scale (0 = very left-wing; 10 = very right-wing). Additionally, environmental identity was also measured on a 10-point-sliding scale (0 = not environmental; 10 = very environmental). [↑](#footnote-ref-1)
